# Would You Rather Be Safe or Free? Motivational and Behavioral Aspects in COVID-19 Mitigation

**DOI:** 10.3389/fpsyg.2021.635406

**Published:** 2021-05-28

**Authors:** Giulio Costantini, Marco Di Sarno, Emanuele Preti, Juliette Richetin, Marco Perugini

**Affiliations:** ^1^Department of Psychology, University of Milan-Bicocca, Milan, Italy; ^2^Personality Disorders Lab, Milan, Italy

**Keywords:** COVID-19, goals, behaviors, principal component analysis, network analysis

## Abstract

This work investigates the relationship between goals and mitigation behaviors during the COVID-19 pandemic in Italy. Study 1 (pilot) identified goals ascribed to following and violating mitigation-related indications. Study 2 investigated the structure of and link between COVID-related goals and behaviors in a large community sample (*N* = 995, 301 men). Our results showed substantial relationships between goals and behaviors. Goals were best described by a bi-dimensional structure (being safe vs. being free), whereas behaviors clustered into a three-component structure (hygiene, distancing, going out). Hierarchical multiple regressions demonstrated the incremental validity of goals in the prediction of behaviors. Network analysis suggested that goals imbued with social content were more directly related to both risky and preventive behaviors. Motivational aspects emerged as important contributors to the organization of behaviors in the context of the COVID-19 pandemic.

## Introduction

The world pandemic of the coronavirus disease 2019 (COVID-19) originated in China at the end of the year, caused by the Severe Acute Respiratory Syndrome Coronavirus 2 (SARS-CoV-2). In early 2020, COVID-19 became an issue of public interest in Italy (Italian Ministry of Health [IMH], 2020). On February 21^st^, 20 days before the World Health Organization (WHO) confirmed the disease to be pandemic, Italy was the first European country to identify a local cluster of SARS-CoV-2. In the absence of effective pharmaceutical measures to contain the infection, the most effective mitigation strategies available were based on social distancing (Fong et al., 2020; Koo et al., 2020; Prem et al., 2020). Being confronted with a rapidly growing number of SARS-CoV-2 cases, Italy was the first European country to impose severe restrictions on the population in an attempt to reduce the increasing diffusion of the virus and the mounting pressure on the national health system (Remuzzi and Remuzzi, 2020). Such restrictions were first implemented in northern regions, the most severely hit by the virus, but were soon extended to the rest of the country. They included strict curfew measures, the lockdown of most commercial and public activities, quarantine for those infected, and limitations concerning public and private social contacts^1^. Such mitigation measures were enforced in Italy in their strictest version until the beginning of May, for approximately 2 months. These measures were called “Phase 1” lockdown, followed by a “Phase 2” in which restrictions were gradually reduced according to the virus diffusion.

From a psychological standpoint, individuals have shown a broad pattern of responses to the diffusion of the virus (e.g., Wang et al., 2020) and the restrictive measures imposed (e.g., Brooks et al., 2020). For example, the pandemic has had consequences for laypersons’ and healthcare professionals’ mental health (Duan and Gang, 2020; Preti et al., 2020a,b; Tan et al., 2020). Besides, given the importance of individual behaviors for the efficacy of mitigation measures, understanding psychological responses to this new and extreme situation has become of utmost importance.

Recent studies suggested relatively high levels of compliance with COVID-mitigation norms in the initial phases of the infection, including social distancing (e.g., social gathering avoidance, curfew) and hygienic practices (e.g., handshake avoidance) (Barari et al., 2020; Li et al., 2020; Van Bavel et al., 2020). However, compliance seemed to be generally higher among women, senior respondents, individuals reporting better physical health (Barari et al., 2020), those who identified more with their nation (Van Bavel et al., 2020), those perceiving the situation to be more controllable as well as more severe (Li et al., 2020), and those viewing realistic threats to their physical and financial safety (Kachanoff et al., 2020).

Individual behaviors, feelings, and cognitions are shaped by individual motives (Perugini and Conner, 2000; Kruglanski et al., 2002, 2015; Costantini et al., 2020). Goals are cognitive representations of outcomes we wish to approach or avoid (Elliot and Fryer, 2008) and represent a subset of motivational concepts with a relevant impact on our life. They thus serve a crucial regulatory function for our behaviors (Heller et al., 2009) both in the short- and long-term (Kruglanski and Szumowska, 2020).

In the health domain, research on goals provides insights into why people engage in health-compromising behaviors and fail or succeed in changing health-related behaviors (e.g., Sheeran et al., 2017). Previous research suggested that understanding health-related behaviors arguably requires a deep knowledge of individuals’ goals. People pursue multiple goals at the time, some of which are incompatible, and health-related behaviors result from the interplay of such goals and their relative importance (Gebhardt, 2006). For instance, it has been shown how healthy behaviors (e.g., physical activity, sexual behaviors) are influenced by several goals, not only related to being healthy and safe but also to social motivations (e.g., having active social lives) or self-presentation (e.g., being appreciated by others) (Gebhardt and Maes, 1998; Martin et al., 2001). Social distancing during COVID-19 pandemic has shown to correlate strongly with conventional health behaviors, suggesting that insights into general health behaviors can also be transferred to investigate mitigation behaviors (Bourassa et al., 2020).

During COVID-19 pandemic, the restrictions imposed arguably challenged the possibility for individuals to pursue personal goals. However, recent research shows that affirming a sense of autonomy, despite restrictions related to COVID-19, increases psychological well-being (Cantarero et al., 2020). Hence, knowledge of personal goals that people ascribe to behaviors related to COVID-19 might be crucial in shaping such behaviors (e.g., through media campaigns). However, little is known regarding the specific motivations that individuals ascribe to COVID-related behaviors and cognitions and how they shape their adherence to COVID-related norms.

The aim of this paper is twofold. First, we aim at identifying a comprehensive set of goals that individuals ascribe not only to adhering but also to violating norms related to COVID-19 mitigation. Second, we aim at investigating the relationship between the identified goals and specific mitigation-related behavioral outcomes. This would allow identifying a set of goals that could be leveraged, for instance, to develop interventions promoting adherence to norms related to COVID-19 and discouraging their violation (e.g., Freitas et al., 2004; Prestwich et al., 2010; Oettingen, 2012; Wieber and Gollwitzer, 2017). The work is articulated in two studies, both carried out in Italy between March and April 2020, during the “Phase-1” lockdown. Both studies received approval by the Ethical Committee in charge.

## Study 1

### Materials and Methods

Study 1 is a pilot study aiming at the identification of a comprehensive set of goals that individuals ascribed to enacting and violating COVID-mitigation norms, using qualitative responses collected during the “Phase-1” lockdown in Italy.

#### Participants

Seventy-seven participants (30 men, 38.9%) with a mean age of 40.1 years (*SD* = 18.1) took part in the study.

#### Materials and Procedure

Before the beginning of the study, participants provided online informed consent. Using Qualtrics^2^, participants read a set of 12 appropriate COVID-mitigation indications (e.g., “stay at home,” “wash hands frequently”), based on directions from the WHO and the Italian Ministry of Health (Italian Ministry of Health [IMH], 2020; e.g., World Health Organization [WHO], 2020a). Then, they were asked to generate 3 to 5 goals that they could pursue by respecting official recommendations and another set of 3 to 5 goals that they could pursue by violating such indications. Goals for respecting vs. violating norms were investigated separately, considering that goals for action and inaction are distinct and not necessarily opposite (Richetin et al., 2011). There were no pre-specified response options: participants wrote free-text goals in each of the two conditions. For the sake of clarity, participants were provided with a brief description of what a goal is (e.g., “something you try to obtain through behaviors”) and what it is not (e.g., “causes or explanations”), as well as with typical examples of goals and non-goals.

Since Study 1 was generative, we could not determine the sample size using power analysis. Our setup required to indicate between 6 and 10 responses (i.e., 3 to 5 for respecting and violating norms, respectively). Thus, seventy-seven participants ensured between 462 and 770 responses. Based on previous studies employing similar methods (Costantini and Perugini, 2018; Costantini et al., 2020), they were deemed enough to convey a sufficient amount of information regarding COVID-related goals.

### Results

Participants generated 540 verbal responses (7.02 on average per participant). Of these, 283 were elicited by the respecting-norms question (i.e., goals related to following indications) and 257 by the violating-norm question (i.e., goals related to not following them). A series of steps were taken to summarize and aggregate the responses. A first analysis was performed to obtain an initial clustering of goals, which was further manually refined to get a final set of goals. Crucially, both steps were performed blind to whether each goal was ascribed to following vs. violating COVID-mitigation indications.

To perform an initial automatic classification, all responses were analyzed with software-based methods. Details of this procedure are presented in Supplementary Materials. An initial set of 80 clusters was identified after this step, including between 2 and 25 responses for each cluster.

We then needed to refine this initial set of clusters manually. Despite the instructions specifying what a goal was, some responses could not be clearly classified as goals (e.g., “I don’t know,” “I trust science”). Furthermore, some clusters included heterogeneous content and needed to be further split into different clusters (e.g., responses “avoid to become infected” and “avoid to infect my relatives” were both in the same cluster). Other clusters included content that overlapped and needed to be merged with one another (e.g., responses “avoid getting infected” and “avoid getting the virus” were in different clusters). Through consensual discussion among the authors, we identified and removed clusters that were regarded as non-goals, merged clusters that were similar in content, and separated clusters that included heterogeneous content. This led to the identification of 33 goal classes. We then reviewed all responses and classified each goal into one of those classes. Some classes referred mainly to consequences of behavior instead of goals (e.g., “getting sick,” “harm others,” “harm the economy”) and were excluded, thus leaving a final set of 22 classes. The final list of classes is presented in Table 1, with the total number of responses falling within each class, and the number of responses split by the original eliciting question (respecting vs. violating norms). Some classes mainly represented responses that had been elicited by the norm-respecting question (e.g., class “containment of the contagion” included 68 responses, all elicited by the norm-respecting question), whereas other classes mainly included responses elicited by the norm-violating question (e.g., class “pursue personal freedom” included 8 responses, 7 of which elicited by the norm-violating question). We tested whether these 22 classes were significantly associated with the eliciting condition, using a chi-square test for independence, with bootstrapped *p*-values. The chi-square tested the null hypothesis of independence between the type of eliciting question and the goal class (i.e., responses in each class were elicited with the same frequency by the two questions of respecting vs. violating norms). Results suggested rejecting the null hypothesis, indicating a significant association between goals and the eliciting condition, χ^2^(21) = 256.35, *p* < 0.001. In other words, goals ascribed to respecting COVID-mitigation indications generally fell into different classes compared to goals ascribed to violating such norms.

**TABLE 1:** Goal classes emerged in Study 1, their frequency, and the number of times they were ascribed to respecting and to violating COVID-mitigation indications.

**Goal class**	**Frequency**	**Respecting norms**	**Violating norms**
Containment of the contagion (e.g., “reduce the probability of contagion,” “stop contagion in my region”)	68	68	0
Protect oneself (e.g., “avoid getting sick,” “staying healthy”)	55	52	3
Protect loved ones (e.g., “safeguard my family,” “take care of my nieces”)	25	21	4
Protect the society (e.g., “safeguard public health,” “help protecting the life of the weaker individuals”)	21	20	1
Getting back to normality (e.g., “reduce the duration of the global pandemic,” “getting back to circulating freely as soon as possible”)	21	19	2
Safeguard the healthcare system (e.g., “avoid the collapse of the national healthcare system,” “reduce overcrowding in hospitals”)	19	19	0
Study/work (e.g., “graduate,” “get back to work”)	24	13	11
Hygiene (e.g., “clean my house,” “clean surfaces”)	9	9	0
Personal growth (e.g., “devote more time to myself,” “do activities I did not have the time to do before”)	10	9	1
Do cultural activities (e.g., “reading,” “go to the cinema”)	14	8	6
Safeguard the economy (e.g., “avoid a further recession,” “avoid economic problems”)	10	8	2
Respect norms and rules (e.g., “respect rules,” “respect others”)	5	5	0
Be an example for others (e.g., “teach other citizens the need for everyday hygiene,” “empower (others)”)	4	4	0
Meet someone (e.g., “meet my friends,” “spend time with my family”)	48	6	42
Do sports (e.g., “go running,” “go to the gym”)	19	5	14
Stay in the open air (e.g., “go out for a walk,” “walk in the nature”)	15	2	13
Develop interpersonal relationships (e.g., “having a social life,” “hug”)	16	5	11
Traveling (e.g., “travel abroad,” “doing a trip for the weekend”)	10	0	10
Pursue personal freedom (e.g., “move freely,” “feel free”)	8	1	7
Getting supplies (e.g., “go out for groceries,” “go to the market”)	6	0	6
Relax, experience positive emotions (e.g., “having fun,” “fight against boredom”)	7	1	6
Live a normal life now (e.g., “being able to scratch my own eyes when they are itchy,” “live my usual life, with the usual routines”)	5	0	5

### Discussion

Study 1 allowed identifying a broad set of goals that individuals ascribe to respecting and to violating COVID-mitigation indications. We used the resulting categorization (i.e., the 22 classes of Table 1) to generate a questionnaire to assess goals related to COVID-19, the *COVID-19 Goal Questionnaire*. In particular, we generated 25 statements, each corresponding to an item of the *Goal Questionnaire*. The 25 statements were based on the original 22 goal classes, but were articulated and rearranged to emphasize competitions between motivations. In particular, items contrasted normality and restrictions (e.g., “Try not to change my life because of the epidemic”), immediate and long-term social gratifications (e.g., “Meet my friends immediately, even if this may involve risks”), health issues and renounces (e.g., “Contain the contagion, even if this may imply important sacrifices”), economic and health-related priorities (e.g., “Protect my economic situation, even at the cost of some risks for my health”), personal health and entertainment (e.g., “Stay safe, even if this means getting bored”), others’ and personal well-being (e.g., “Give more importance to my well-being than to the general well-being”), and respecting *versus* violating norms and rules (e.g., “Do something for the sake of breaking the rules”).

## Study 2

The main aim of Study 2 was to investigate connections between goals and behaviors related to COVID-19. The preregistered hypothesis was a significant correlation between the leading principal components of COVID-related goals and behaviors relevant for COVID-19 mitigation, reflecting a general connection between goals and behaviors.

Several additional exploratory analyses were performed to further deepen the relationships among specific types of goals and behaviors. First, principal component analysis (PCA) was employed to summarize the main dimensions of variance of goals and of behaviors related to COVID-19 with a limited number of components (Comrey and Lee, 1992). Second, hierarchical regression was employed to assess the relative contribution of different goals in predicting behavior. Our main interest was in deepening the relationship between goals and behaviors. Still, we considered other variables relevant to COVID-19 to investigate goals’ incremental validity. Demographic variables are known to be related to mitigation behaviors and adhesion to norms (e.g., Barari et al., 2020). This also seems to depend on the fact that individuals have different vulnerabilities to COVID-19 according to their age and gender, with the elderly and males being more vulnerable (Aschwanden et al., 2020; Jin et al., 2020). Furthermore, since at the time of the study, the SARS-CoV-2 was unevenly spread throughout Italy, we considered the potential effect of respondents’ current location by splitting Italian regions into low-risk (southern regions, with a lower prevalence of cases), medium risk (Piedmont, Veneto, and Emilia-Romagna), and high-risk (Lombardy). A detailed distribution of our sample by location/region is in Supplementary Table 1. We also reasoned that individuals might have different levels of concern and knowledge about COVID-19: Correct information is a necessary (though not sufficient) element to promote healthy behaviors (Tan et al., 2015; Wu and Shen, 2021). Furthermore, whereas some individuals might have been directly affected by COVID-19, others might have only had an indirect experience of the virus. Health behavior is shaped by fear of negative consequences (e.g., Rogers, 1975, 1983). All these elements might result in different behaviors, irrespective of personal goals. We thus inspected whether goals would provide additional information about behaviors related to COVID-19, even after controlling for age, gender, location, education, knowledge, concerns, and personal impact of COVID-19.

The aforementioned analyses focused on the pattern of relationships among principal components of behaviors and goals. We thus employed psychometric network analysis (Epskamp et al., 2018c; Costantini et al., 2019) to further investigate the pattern of relationships involving specific goals and behaviors, beyond what could be captured by broader components.

### Materials and Methods

#### Participants and Procedure

This study is part of a project on personality, motivation, and COVID-related behaviors in which we collected data from two samples of participants. Both samples completed a common set of measures: for the purpose of this paper, only those data were analyzed. The two data collections can be found in two independent preregistrations^3^. However, both studies were preregistered with the same hypothesis, a correlation between the first principal components of goals and of behaviors, which is the focus of this work. Data were collected between April 17th and 30th, 2020.

One thousand six hundred seventy-five participants began the online survey using Qualtrics, lasting around 40 min. Inclusion criteria were being in Italy at the time of the questionnaire completion and being above 18 years old. An item to detect careless responding was included in the questionnaire to filter out participants (Self-Reported Single-Item “Use-Me” indicator), as recommended by Meade and Craig (2012). Six hundred and eighty participants were excluded from the analyses, either because they indicated careless responding to the “Use-Me” indictor (*N* = 40), or because they quit the questionnaire before completing the indicator (*N* = 640). The final sample was *N* = 995, 301 men, 688 women, two who self-identified as transsexual, and four who self-identified as “other.” The age range was 18–79, *M*_*age*_ = 32.88, *SD* = 13.05. There were no missing values^4^.

In our preregistration, we planned for a sample size of at least *N* = 300, which would allow detecting a correlation as small as *r* = 0.14 with a power of 0.80 at the conventional alpha level of 0.05 in a one-tailed test. However, we did not define an *a priori* stopping rule, aiming for a sample size as large as possible before running analyses. The final sample of *N* = 995 allows detecting a correlation as small as *r* = 0.08 under the same conditions.

#### Measures

Before the beginning of the study, participants provided online informed consent. All participants completed the following measures, in the following order:

*Demographic Information Questionnaire* included gender, age, education, location, and occupation (student status). Participants also provided information regarding their personal experience with COVID-19 (see Table 2).

*COVID-19 Knowledge Questionnaire*. Participants completed ten questions investigating their knowledge of COVID-19 (e.g., “COVID-19 always causes symptoms”) with a three-option response format (*true*/*false*/*I don’t know*), and only one option being correct. Correct knowledge was determined based on what was considered to be correct by WHO and the Italian Ministry of Health at that point in time. Correct answers were coded as 1, and a sum score was computed, with higher scores indicating sounder knowledge.

*COVID-19 Goal Questionnaire.* Participants rated the personal importance of 25 goals related to COVID-19, using a five-point scale ranging from 1 (*not at all important*) to 5 (*extremely important*). This scale was derived from Study 1 (see paragraph 2.3).

*COVID-19 Behaviors Questionnaire.* Participants indicated the frequency with which they enacted each of 18 behaviors on a scale from 1 (*Never or almost never*) to 5 (*Always or almost always*). The scale was adapted from the directions of the WHO and the Italian Ministry of Health. Some behaviors corresponded to rules/laws emitted during the lockdown (e.g., “Avoid gatherings”). Some were recommendations with a more personal value (e.g., “wash hands frequently”), and some represented subtle transgressions of the rules (e.g., “Leave home very often to do sports outdoor”).

*COVID-19 Concern Questionnaire* (Conway et al., 2020). The scale is a six-item measure assessing concern for the coronavirus disease (e.g., “Thinking about the coronavirus (COVID-19) makes me feel threatened”) with a five-point scale ranging from 1 (*completely false for me*) to 5 (*completely true for me*). This and the following scale were translated into Italian through back-translation. Cronbach’s alpha for the scale was 0.86 in our sample.

*COVID-19 Impacts Questionnaire* (Conway et al., 2020). The measure is a nine-item questionnaire assessing the financial and psychological impact of the coronavirus disease and the difficulty in obtaining necessary resources during the lockdown. Participants used a five-point Likert scale ranging from 1 (*completely false for me*) to 5 (*completely true for me*). Cronbach’s alphas in our sample were 0.77 (resource scale), 0.80 (psychological scale), and 0.92 (financial scale).

#### Data Analyses

All analyses were performed using R (R Core Team [RCT], 2020). We used the packages *psych* (Revelle, 2019) and *GPArotation* (Bernaards and Jennrich, 2005) to perform PCA on both goals and behaviors. The preregistered hypothesis of a general relationship between goals and behaviors was tested by extracting the first principal component from each questionnaire separately and by inspecting the correlation between these two component scores. The structure of the *COVID-19 Goal Questionnaire* was then further examined using PCA. Criteria for selecting the number of components included eigenvalues higher than 1, exploration of the scree-plot, and parallel analysis (Horn, 1965; Comrey and Lee, 1992; Hayton et al., 2004). The extracted components were then rotated using *oblimin*, and component scores were saved to use in subsequent analyses. A similar analysis was performed for exploring the structure of the *COVID-19 Behaviors Questionnaire*. We then examined the correlations among specific components of goals and behaviors.

Hierarchical multiple regression models were carried out to investigate the incremental effects of goals in predicting behaviors (component scores). In the first step, demographic variables were included as predictors (gender, age, education, location); in the second step, COVID-19 knowledge, impacts, and concern scales were added to the models; in the third and last step, goal classes were also included.

The relationship among specific behaviors and goals was explored using network analysis in the form of a Gaussian Graphical Model (Costantini et al., 2015, 2019; Epskamp et al., 2018c). We estimated the network using the graphical lasso algorithm, based on polychoric correlations, as implemented in the R package qgraph (Epskamp et al., 2012, 2018b). The lasso regularization parameter was selected through the *Extended Bayesian Information Criterion* (Chen and Chen, 2008; Foygel and Drton, 2010), with the hyperparameter gamma set to the default value of 0.50 (Epskamp and Fried, 2018). We examined the centrality of each node (Freeman, 1978). Since our interest was mainly into connections between goals and behaviors, as opposed to connections among goals and behaviors separately, we focused on *bridge strength centrality*, which quantifies the amount of connectivity of a node with nodes of another group of nodes (Jones et al., 2019), using the networktools package (Jones, 2020). To control the replicability of our results, we also computed the bootstrap-based *correlation stability coefficient* (*CS*-coefficient) of bridge centrality using package bootnet (Epskamp et al., 2018a). The CS-coefficient corresponds to the maximum proportion of cases that could be dropped, such that the correlation between the original bridge strength and the one computed in the reduced sample would still be larger than *r* = 0.70 with 95% probability. Values of the CS-coefficient larger than 0.25 indicate sufficient stability, whereas values of 0.50 or larger are preferable.

### Results

#### Descriptive Statistics

Around one-third of our participants were students (*N* = 364, 36%), and only 36 of them attended high school. Around 53% of the sample (*N* = 525) had a university level of education or above, whereas 41% of our participants (*N* = 407) had achieved a high-school diploma, 6% a secondary school (*N* = 59), and four participants had a primary-school level of education (< 1%). Many participants (*N* = 549, 55%) declared to know at least one person (e.g., nurse, doctor) employed in the National Health System against COVID-19.

Table 2 presents means and standard deviations for scales of concern and impacts, COVID-19 knowledge, and COVID-related symptoms and tests. Participants had a good knowledge of the disease on average, and only a handful had been diagnosed with the disease (*N* = 5).

**TABLE 2:** COVID-related sample characteristics.

	*M* (*SD*)	Range
Financial impact	3.19 (1.34)	1–5
Resource impact	2.10 (0.92)	1–5
Psychological impact	2.62 (1.02)	1–5
COVID concern	3.60 (0.85)	1–5
COVID knowledge^*a*^	8.53 (1.18)	3–10

	**“…*experienced COVID-compatible***	
	***symptoms?*”**	
	**Yes - *N* (%)**	**No - *N* (%)**

“*Have you*…*?*”	90 (9%)	905 (91%)
“*Has any of your family members*…”	135 (14%)	860 (86%)
“*Has any of your friends/colleagues*…”	384 (39%)	611 (61%)

	**“…*been tested (through swab)***	
	***for COVID-19?*”**	
	**Yes - *N* (%)**	**No - *N* (%)**

“*Have you*…*?*”	33 (3%)	962 (97%)
“*Has any of your family members*…”	120 (12%)	875 (88%)
“*Has any of your friends/colleagues*…”	266 (27%)	729 (73%)

	**“…*been diagnosed with COVID-19***	
	**i.e., positive swab)?” - *N***	

“*Have you*…*?*”	5	
“*Has any of your family members*…”	46	
“*Has any of your friends/colleagues*…”	194	

*M, mean; SD, standard deviation. ^*a*^Sum of correct information regarding COVID-19 (10 questions).*

#### Behaviors and Goals

Following the preregistered hypothesis plan, we extracted the first principal component from goals and behaviors separately and retained all items with loadings < | 0.30| (only two behaviors were discarded). The goal component, explaining 33% of variance, had positive loadings by goals connected to pursuing personal freedom despite risks connected to COVID-19 and negative loadings by goals connected to pursuing security despite personal freedom (for details, see Supplementary Table 2). The behavioral component (21% of variance) had positive loadings by behaviors connected to social distancing and hygiene and negative loadings by behaviors connected to leaving the house (see Supplementary Table 3). The correlation between the two was *r* = −0.41, *p* < 0.001 (similar analyses considering the two preregistered subsamples separately yielded nearly identical results, *r*s = −0.38 and −0.43, *p*s < 0.001), indicating as hypothesized a substantial general connection between goals and behaviors.

To further explore the relationships among goals and behaviors, respectively, we examined in detail the structure of each questionnaire. Parallel analysis and scree-test suggested a two-component solution for the *COVID-19 Goals Questionnaire*. The two components explained 40% of the cumulative variance and were correlated −0.50, corroborating the use of an oblique rotation procedure. Table 3 presents loadings for the two-component solution: The two latent factors were named “safe” (G1) and “free” (G2), the first one reflecting goals to protect oneself and others from the virus, and the second representing goals to be free from restrictions. Two items, #9 and #19, showed somewhat smaller loadings and were both related to preserving the economy.

**TABLE 3:** PCA loadings for the COVID-19 goal questionnaire.

Item		Two-component solution	
Italian version	English translation	G1 (safe)	G2 (free)
*1. Contenere il contagio, anche se ciò può significare delle rinunce importanti*	*1. Contain the contagion, even if this may imply important sacrifices*	0.61	
*7. Salvaguardare il sistema sanitario (es. prevenire o ridurre la pressione sulle strutture sanitarie, evitarne il collasso), anche se ciò significa qualche danno economico in più*	*7. Safeguard the healthcare system (e.g., prevent or reduce pressure on health care facilities, avoid their collapse), even if this means extra economic damage*	0.55	
*10. Stare al sicuro (es. non-correre rischi, non-mettersi in pericolo, stare in salute, salvaguardarsi), anche se ciò significa annoiarsi*	*10. Stay safe (e.g., don’t take risks, don’t put yourself in danger, stay healthy, protect yourself), even if this means getting bored*	0.60	
*11. Tornare a condurre una vita normale solo quando si sarà più sicuri*	*11. Go back to leading a normal life only once it will be safer*	0.75	
*13. Proteggere la mia salute, anche se questo significa per me una perdita economica*	*13. Protect my health, even if this means financial losses for me*	0.71	
*14. Tornare a poter vedere i miei cari (es. genitori, figli, parenti, fidanzati, ecc.) solo una volta che si è più sicuri*	*14. See my loved ones again (e.g., parents, children, relatives, partners, etc.) only once it is safer*	0.70	
*17. Stare al sicuro anche rinunciando a procurarmi ciò che vorrei*	*17. Stay safe, even if giving up getting what I want*	0.69	
*20. Proteggere la collettività, gli altri in generale, anche a scapito del mio benessere immediato (Far sì che gli altri stiano al sicuro, evitare che si ammalino)*	*20. Protect the community, others in general, even at the expense of my immediate well-being (ensure others are safe, prevent them from getting sick)*	0.58	
*21. Rispettare norme e regole (es. rispettare la legge, le indicazioni del ministero della salute etc.)*	*21. Respect norms and rules (e.g., respect the law, the directions from the Ministry of Health, etc.)*	0.61	
*22. Proteggere i miei cari (genitori, figli, parenti, fidanzati, ecc.), anche a scapito del mio benessere immediato (Far sì che i miei cari stiano al sicuro, evitare che si ammalino)*	*22. Protect my loved ones (parents, children, relatives, partners, etc.), even at the expense of my immediate well-being (ensure my loved ones are safe, prevent them from getting sick)*	0.59	
*24. Dare il buon esempio agli altri*	*24. Set a good example to others*	0.61	
*25. Tornare a poter vedere i miei amici solo una volta che si è più sicuri.*	*25. See my friends again only once it is safer*	0.71	
*2. Incontrare subito i miei cari (es. genitori, figli, parenti, fidanzati, ecc.), anche se ciò potrebbe comportare dei rischi*	*2. Meet my loved ones immediately (e.g., parents, children, relatives, partners, etc.), even if this may involve risks*		0.65
*3. Cercare di non-modificare la mia vita a causa dell’epidemia*	*3. Try not to change my life because of the epidemic*		0.61
*4. Incontrare subito i miei amici, anche se ciò potrebbe comportare dei rischi*	*4. Meet my friends immediately, even if this may involve risks*		0.78
*5. Socializzare ora, anche correndo qualche rischio (es. incontrare persone, conoscere persone nuove, avere contatto fisico)*	*5. Socialize now, even taking some risks (e.g., meet people, get to know new people, have physical contact)*		0.78
*6. Sentirmi libero/a di fare ciò che voglio (fare ciò che mi va, senza dovermi giustificare con nessuno)*	*6. Feel free to do what I want (do what I want, without having to justify myself to anyone)*		0.68
*8. Ridurre ora i disagi dovuti alle restrizioni, anche se significa correre qualche rischio in più*	*8. Reduce the inconvenience of restrictions now, even if it means taking a few more risks*		0.58
*15. Divertirmi, anche se ciò significa correre qualche rischio in più*	*15. Have fun, even if it means taking a few more risks*		0.72
*16. Fare le cose che sono importanti per me, anche se potrebbero comportare dei rischi per gli altri*	*16. Do the things that are important to me, even though they may involve risks for others*		0.61
*9. Proteggere la mia situazione economica, anche a costo di qualche rischio per la salute*	*9. Protect my economic situation, even at the cost of some risks for my health*	−0.29	0.34
*12. Fare qualcosa per il gusto di trasgredire le regole*	*12. Do something for the sake of breaking the rules*		0.47
*18. Stare all’aria aperta (es. fare un giro in città o al parco, passeggiare, fare sport, etc.), anche se potrebbe comportare qualche rischio di più*	*18. Be outdoor (e.g., take a walk in the city or park, walk, do sports, etc.), although it may involve some extra risk*	−0.32	0.48
*19. Salvaguardare il sistema economico (es. evitare la recessione, minimizzare i danni economici), anche se ciò significa correre qualche rischio sanitario di più*	*19. Safeguard the economic system (e.g., avoid recession, minimize economic damage), even if this means taking some more risks for health*	−0.27	0.31
*23. Dare più importanza al mio benessere che a quello generale*	*23. Give more importance to my well-being than to the general well-being*		0.40

*PC, principal component; loadings below 0.20 are omitted; secondary loadings are in gray.*

As to behaviors, parallel analysis and scree-test suggested a three-component solution for the measure, explaining 43% of the cumulative variance. Components were correlated between −0.04 to −0.22 (details in Table 4). Loadings are presented in Table 5. We named the three components “hygiene” (B1), “social distancing” (B2), and “going out” (B3), respectively. The first reflected hygiene-focused behaviors, such as washing hands. The second included items assessing the tendency to keep safety distance in social relationships. The third component comprised norm-incongruent behaviors, specifically related to going out and/or meeting people.

**TABLE 4:** Pearson correlations among latent scores, Conway scales, COVID knowledge, age, and education.

	1.G1	2.G2	3.B1	4.B2	5.B3	6.Age	7.Edu	8.Knw	9.RI	10.PI	11.FI	12.C
1. G1safe	**0.86**											
2. G2free	−0.50***	**0.84**										
3. B1hygiene	0.28***	−0.12***	**0.74**									
4. B2distance	0.25***	−0.25***	0.19***	**0.66**								
5. B3out	−0.30***	0.38***	0.04	−0.22***	**0.66**							
6. Age	–0.02	–0.05	0.24***	0.11***	0.04	–						
7. Education	–0.05	0.09**	–0.05	0.14***	–0.05	–0.01	–					
8. Knowledge	0.16***	−0.12***	0.17***	0.14***	−0.10**	0.10**	0.12***	–				
9. R. Impact	−0.13***	0.11**	0.02	–0.05	0.02	0.07*	–0.06	–0.05	**0.77**			
10. P. Impact	0.00	0.09**	0.06*	–0.04	0.02	−0.08*	–0.05	0.04	0.15***	**0.80**		
11. F. Impact	–0.05	0.05	0.05	0.04	0.01	0.00	−0.09**	–0.03	0.23***	0.13***	**0.92**	
12. Concern	0.43***	−0.23***	0.34***	0.14***	−0.19***	0.13***	–0.03	0.15***	0.09**	0.23***	0.08*	**0.86**

Cronbach’s alphas are in bold on the diagonal (when applicable): for goals and behaviors, items are considered to load on the scale with the highest factor loading. *p < 0.05; **p < 0.01; ***p < 0.001.

**TABLE 5:** PCA loadings for the COVID-19 behaviors questionnaire.

Item		Three-component solution		
Italian version	English translation	B1 (hygiene)	B2 (social distancing)	B3 (going out)
*7. Lavare frequentemente le mani*	*7. Wash my hands frequently*	0.75		
*8. Pulire le mani con gel o salviette igienizzanti, quando non si dispone di acqua e sapone*	*8. Clean my hands with gel or sanitizing wipes when I do not have soap and water*	0.79		
*14. Non-toccarsi occhi, naso e bocca con le mani, senza averle lavate prima*	*14. Do not touch my eyes, nose, and mouth with hands without washing them in advance*	0.69		
*17. Pulire le superfici con disinfettanti a base di cloro o alcol*	*17. Clean surfaces with chlorine or alcohol-based disinfectants*	0.72		
*13. Igiene respiratoria (starnutire e/o tossire in un fazzoletto evitando il contatto delle mani con le secrezioni respiratorie)*	*13. Respiratory hygiene (sneeze and/or cough in a handkerchief avoiding hand contact with respiratory secretions)*	0.50	0.26	
*16. Evitare l’uso promiscuo di bottiglie e bicchieri*	*16. Avoid sharing bottles or glasses*	0.40	0.37	
*18. Usare i guanti e/o la mascherina fuori casa (incluse protezioni alternative come sciarpe o bandane)*	*18. Use gloves and/or mask when outside (including alternative protections such as scarves or bandanas)*	0.42		
*9. Evitare assembramenti di persone*	*9. Avoid people gatherings*		0.71	
*10. Evitare di creare occasioni di incontro in casa o fuori, con amici, parenti e/o vicini*	*10. Avoid creating opportunities to meet at home or outside, with friends, relatives and/or neighbors*		0.60	
*11. Mantenere, nei contatti sociali, una distanza interpersonale di almeno un metro (escluse le persone con cui si convive)*	*11. Maintain, in social contacts, an interpersonal distance of at least one meter (excluding people with whom you live together).*		0.69	
*12. Evitare abbracci e/o strette di mano (tranne che con le persone con cui si convive)*	*12. Avoid hugs and/or handshakes (except with people you live with)*		0.70	
*15. Evitare il contatto ravvicinato con persone che soffrono di infezioni respiratorie acute*	*15. Avoid close contact with people suffering from acute respiratory infections*		0.59	
*1. Restare a casa, uscire di casa solo per esigenze lavorative, motivi di salute e necessità*	*1. Stay at home, leave home only for work, health and necessity reasons*	−0.20	0.36	
*2. Uscire di casa (ad es., per fare la spesa o andare in farmacia), anche se non-strettamente necessario*	*2. Leave the house (e.g., to go shopping or to the pharmacy), even if not strictly necessary*			0.69
*3. Uscire di casa per motivi consentiti dalla normativa e approfittarne per allontanarsi e fare due passi*	*3. Leave home for reasons permitted by law and take advantage of it to get away and go for a walk*			0.71
*4. Uscire molto spesso di casa per fare sport all’aperto*	*4. Go out of the house very often for outdoor sports*			0.69
*5. Cercare delle ragioni qualsiasi per uscire di casa, anche se non-veramente necessario*	*5. Look for any reason to leave the house, even if it’s not really necessary*			0.69
*6. Incontrare persone (escluse le persone con cui si convive)*	*6. Meet people (excluding people you live with)*		−0.23	0.45

*PC, principal component; loadings below 0.20 are omitted; secondary loadings are in gray.*

Table 6 presents descriptive statistics of goals and behaviors and differences by gender (men vs. women) and by location. Women scored higher on G1 “safe” and lower on G2 “free.” As for behaviors, women were higher on focused hygiene (B1) and lower on going-out behaviors (B3) on average. There was no gender difference in social distancing (B2). ANOVAs indicated there were significant differences in G1 “safe” based on the location of participants, with higher scores in low-risk regions, followed by Lombardy and medium/high-risk regions. At the same time, Lombardy had the lowest score on the “going out” scale.

**TABLE 6:** Descriptive statistics of goals and behaviors.

	*M* (*SD*)	Range	S (K)	Gender differences (*df* = 987)		Differences by location (*df = 2,992)*		
				*M* (*SD*)_*men*_	*M* (*SD*)_*women*_	*M* (*SD*)_*high*_	*M* (*SD*)_*medium*_	*M* (*SD*)_*low*_
G1safe	4.27 (0.53)	1.75–5	−0.89 (1.06)	**4.15** (0.59)	**4.31** (0.49)	**4.27** (0.52)	**4.17** (0.58)	**4.34** (0.49)
				*t* = −4.41***; *d* = 0.30		*F* = 6.77**; η^2^ = 0.13		
G2free	1.97 (0.58)	1–4.77	0.81 (0.78)	**2.08** (0.64)	**1.91** (0.54)	**1.95** (0.58)	**2.01** (0.56)	**1.96** (0.59)
				*t* = 4.30***; *d* = 0.30		*F* = 0.70; η^2^ = 0.00		
B1hygiene	4.07 (0.69)	1.57–5	−0.76 (0.31)	**3.97** (0.75)	**4.11** (0.66)	**4.07** (0.68)	**4.00** (0.72)	**4.13** (0.68)
				*t* = 2.96**; *d* = 0.20		*F* = 2.50; η^2^ = 0.00		
B2distance	4.68 (0.50)	1.33–5	−2.42 (7.59)	**4.66** (0.51)	**4.68** (0.50)	**4.69** (0.46)	**4.67** (0.53)	**4.65** (0.56)
				*t* = −0.81; *d* = 0.05		*F* = 0.72; η^2^ = 0.00		
B3out	1.22 (0.39)	1–3.60	2.82 (9.82)	**1.27** (0.44)	**1.20** (0.36)	**1.19** (0.31)	**1.27** (0.47)	**1.24** (0.41)
				*t* = 2.55*; *d* = 0.18		*F* = 3.41*: η^2^ = 0.01		

M, mean; SD, standard deviation; S, skewness; K, kurtosis. *d*, Cohen’s *d* (effect size). η^2^, eta-squared (effect size). For these analyses only, goal and behavior scales were computed as mean scores of the items with primary loadings on them, to facilitate scores’ interpretation. Descriptive statistics and differences by location are computed on the whole sample (*N* = 995); gender differences are computed only between men and women (*trans* and other are not included; *N* = 989). *p < 0.05; **p < 0.01; ***p < 0.001.

Table 4 presents Pearson correlations among specific classes of goals and behaviors and knowledge, concern, and impact related to COVID-19. Given deviations from normality, Spearman rank-order correlations were also computed, with negligible differences (results are available upon request from the corresponding author). G1 “safe” correlated positively with B1 “hygiene” and B2 “distance,” but negatively with B3 “going out.” Goals to be free (G2), on the contrary, were positively correlated with B3 “going out,” and negatively with the other two composite behaviors (especially B2 “distance”). Behaviors were also significantly correlated with COVID-19 concern, and to a lower extent, with knowledge, age, and education. Correlations between behaviors and impact scales were trivial in magnitude.

#### Incremental Effect of Goals and Behaviors

Results from hierarchical multiple regressions predicting the three behavior classes identified through PCA are presented in Table 7. Goals always explained a significant portion of variance above and beyond demographic variables, knowledge, impacts, and concern for COVID. Both goal classes were uniquely related to each of the three behavioral components in expected directions, with the only exception of goal class “free” that did not significantly predict hygiene (B1). COVID-19 knowledge remained positively associated with hygiene (B1) and social distancing (B2) after accounting for goals, but its association with “going out” (B3) became non-significant in the last step. After accounting for goals, concern for COVID-19 still reduced the tendency to go out (B3) and increased the enactment of focused hygiene (B1). On the contrary, its unique effect on social distancing (B2) became non-significant. Being in higher-risk regions only predicted reduced going out (B3) across regressions. Finally, age remained positively associated with all three behavior classes after accounting for goals. Older participants were more likely to enact hygienic practices (B1) and keep social distance (B2), but also to go out (B3).

**TABLE 7:** Predicting behaviors: Incremental effects of goals.

DV	IV	Step 1				Step 2				Step 3			
		β	*SE*	*t*	*CI*	β	*SE*	*t*	*CI*	β	*SE*	*t*	*CI*
**B1 Hygiene**	Gender	0.12	0.03	4.02***	[0.06, 0.19]	0.06	0.03	2.06*	[0.00, 0.12]	0.06	0.03	1.86	[0.00, 0.11]
	Age	0.26	0.03	8.37***	[0.20, 0.32]	0.20	0.03	6.71***	[0.14, 0.26]	0.22	0.03	7.37***	[0.16, 0.28]
	Education	−0.04	0.03	−1.40	[−0.10, 0.02]	−0.04	0.03	−1.52	[−0.10, 0.01]	−0.05	0.03	−1.60	[−0.10, 0.01]
	Location	−0.02	0.03	−0.68	[−0.08, 0.04]	−0.01	0.03	−0.33	[−0.07, 0.05]	0.00	0.03	−0.20	[−0.06, 0.05]
	Knowledge					0.11	0.03	3.66***	[0.05, 0.17]	0.09	0.03	3.15**	[0.03, 0.15]
	R. Impact					−0.02	0.03	−0.64	[−0.08, 0.04]	0.00	0.03	0.08	[−0.06, 0.06]
	P. Impact					0.00	0.03	−0.01	[−0.06, 0.05]	0.01	0.03	0.36	[−0.05, 0.07]
	F. Impact					0.03	0.03	0.92	[−0.03, 0.09]	0.03	0.03	1.15	[−0.02, 0.09]
	Concern					0.29	0.03	9.20***	[0.22, 0.35]	0.21	0.03	6.22***	[0.14, 0.28]
	G1safe									0.20	0.04	5.42***	[0.13, 0.27]
	G2free									0.06	0.03	1.71	[−0.01, 0.12]

		*R*^2^ = 0.07; *F*(4,984) = 20.20***				*R*^2^ = 0.17; Δ*R*^2^ = 0.10; *F*(5) = 24.09***				*R*^2^ = 0.20; Δ*R*^2^ = 0.03; *F*(2) = 14.98***			

**B2 Social distancing**	Gender	0.05	0.03	1.48	[−0.01, 0.11]	0.02	0.03	0.79	[−0.04, 0.09]	0.00	0.03	0.09	[−0.06, 0.06]
	Age	0.12	0.03	3.77***	[0.05, 0.18]	0.09	0.03	2.78**	[0.03, 0.15]	0.10	0.03	3.10**	[0.04, 0.16]
	Education	0.14	0.03	4.48***	[0.07, 0.20]	0.13	0.03	4.21***	[0.07, 0.19]	0.12	0.03	3.80***	[0.06, 0.18]
	Location	0.00	0.03	−0.01	[−0.06, 0.06]	0.00	0.03	0.07	[−0.06, 0.06]	0.00	0.03	0.08	[−0.06, 0.06]
	Knowledge					0.10	0.03	3.23**	[0.04, 0.16]	0.08	0.03	2.48*	[0.02, 0.14]
	R. Impact					−0.06	0.03	−1.82	[−0.12, 0.00]	−0.02	0.03	−0.73	[−0.09, 0.04]
	P. Impact					−0.06	0.03	−1.83	[−0.12, 0.00]	−0.02	0.03	−0.75	[−0.09, 0.04]
	F. Impact					0.07	0.03	2.18*	[0.01, 0.13]	0.08	0.03	2.51*	[0.02, 0.14]
	Concern					0.12	0.03	3.66***	[0.06, 0.19]	0.02	0.03	0.45	[−0.05, 0.09]
	G1safe									0.15	0.04	4.02***	[0.08, 0.23]
	G2free									−0.14	0.03	−4.06***	[−0.21, −0.07]

		*R*^2^ = 0.03; *F*(4,984) = 8.72***				*R*^2^ = 0.07; Δ*R*^2^ = 0.04; *F*(5) = 7.57***				*R*^2^ = 0.12; Δ*R*^2^ = 0.05; *F*(2) = 29.22***			

**B3 Going out**	Gender	−0.09	0.03	−2.78**	[−0.15, −0.03]	−0.06	0.03	−1.73	[−0.12, 0.01]	−0.02	0.03	−0.68	[−0.08, 0.04]
	Age	0.03	0.03	1.02	[−0.03, 0.10]	0.08	0.03	2.38*	[0.01, 0.14]	0.08	0.03	2.57*	[0.02, 0.14]
	Education	−0.04	0.03	−1.23	[−0.10, 0.02]	−0.03	0.03	−1.00	[−0.09, 0.03]	−0.00	0.03	−0.16	[−0.06, 0.05]
	Location	−0.08	0.03	−2.61**	[−0.15, −0.02]	−0.09	0.03	−2.86**	[−0.15, −0.03]	−0.09	0.03	−2.96**	[−0.15, −0.03]
	Knowledge					−0.07	0.03	−2.19*	[−0.13, −0.01]	−0.04	0.03	−1.26	[−0.10, 0.02]
	R. Impact					0.02	0.03	0.50	[−0.05, 0.08]	−0.03	0.03	−0.92	[−0.09, 0.03]
	P. Impact					0.07	0.03	2.31*	[0.01, 0.14]	0.02	0.03	0.68	[−0.04, 0.08]
	F. Impact					0.01	0.03	0.34	[−0.05, 0.07]	0.00	0.03	0.06	[−0.06, 0.06]
	Concern					−0.02	0.03	−6.05***	[−0.27, −0.14]	−0.08	0.03	−2.25*	[−0.15, −0.01]
	G1safe									−0.10	0.04	−2.76**	[−0.18, −0.03]
	G2free									0.31	0.03	9.13***	[0.25, 0.38]

		*R*^2^ = 0.02; *F*(4,984) = 4.45**				*R*^2^ = 0.06; Δ*R*^2^ = 0.04; *F*(5) = 10.85***				*R*^2^ = 0.18; Δ*R*^2^ = 0.12; *F*(2) = 70.25***			

DV, dependent variable; IV, independent variables; SE, standard error; CI, 95% confidence intervals. All variables are standardized; Models exclude participants self-identified as *trans* or *other* and are computed on data from men and women (*N* = 989). Location = coded as 0 for low risk regions, 1 for medium-high risk regions, 2 for high-risk regions (i.e., Lombardy). *p < 0.05; **p < 0.01; ***p < 0.001.

#### Network Analyses of Goals and Behaviors

**FIGURE 1:**
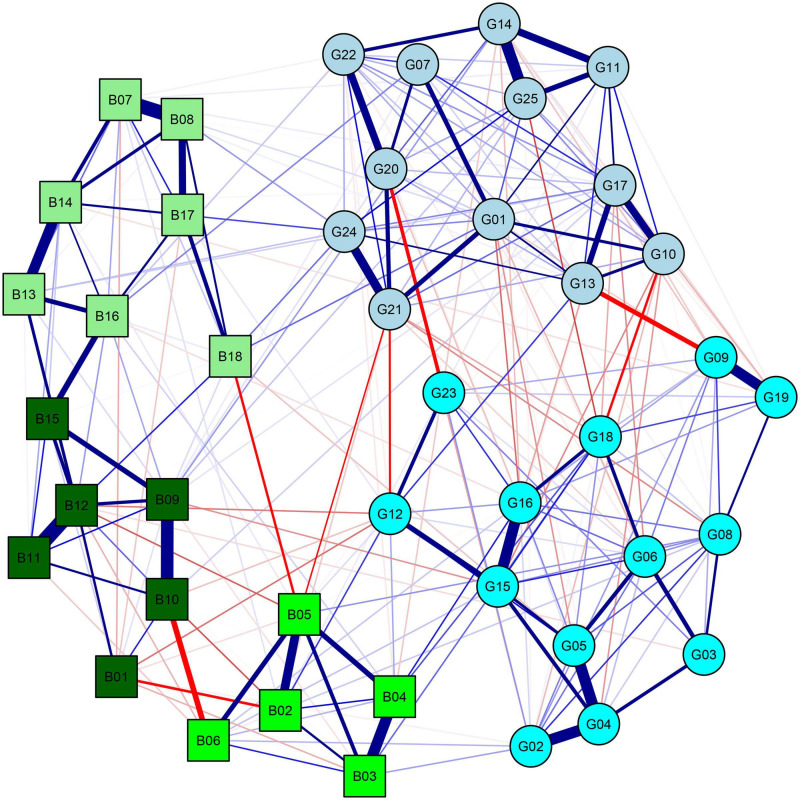
Network of goals and behaviors. Blue and red lines correspond to positive and negative relationships, respectively. Circles correspond to goals, and squared correspond to behaviors. A color was assigned to each behavior and goal according to its highest loadings in the PCA. See Tables 3, 5 for specific goals and behaviors and details about the PCA.

**FIGURE 2:**
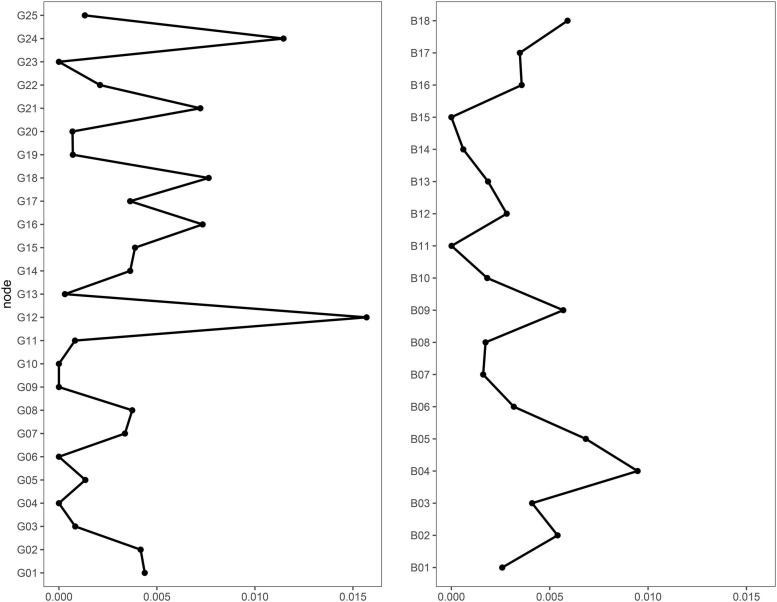
Bridge strength centrality (on the *x* axis) of goals and behaviors. See Tables 3, 5 for specific goals and behaviors and their corresponding number.

We further explored connections among goals and behaviors more specifically (i.e., at the item level) using network analysis. In the resulting network (Figure 1), nodes were divided into two main clusters, one including goals and the other one behaviors. Furthermore, nodes grouped according to the component structure previously identified. The CS-coefficient for bridge Strength was 0.45, thus indicating more than sufficient stability. There were several connections among nodes of the two clusters. However, not all goals entertained similarly strong connections with behaviors (Figure 2). Some goals (#4, #6, #9, #10, #23) were not directly connected to any behavior. Interestingly, two goals showed much larger connections with behaviors than all other nodes, namely goal #12 (“Do something for the sake of breaking the rules”) and goal #24 (“Set a good example to others”).

Goal #12 showed the strongest positive connections to behaviors #2 and #4, which were both related to leaving home in a period in which this was not allowed, and the strongest negative connections to behaviors #1 and #12, which were related to staying at home and avoiding personal contact. This result indicates that the motivation of simply breaking the rules might exert a unique influence on behaviors related to COVID-19. Goal #24 showed the strongest positive connections to behaviors #8, #17, and #18, which were related to COVID-mitigation actions (respectively, cleaning hands, cleaning surfaces, and using masks and gloves). In contrast, it did not show any negative connection with behaviors. This pattern suggests that the desire to be an example to others might directly motivate protective behaviors.

### Discussion

The main aim of Study 2 was to investigate the connection between goals identified in Study 1 and COVID-mitigation behaviors. Results supported the hypothesis of a significant relationship between goals and behaviors in general. Still, further exploration of the structure of goals and behaviors and their connections provided the most interesting insights. Principal component analyses of goals revealed that they conformed to a bi-dimensional structure. The first component represented a general tendency to prioritize safety above other instances (e.g., fun, freedom, immediate well-being, financial issues, need to see beloved people). The second component included goals related to the prioritization of those instances over risk-safeguarding. This latter component also included somewhat antagonistic goals (e.g., breaking the rules, giving more importance to one’s well-being), as opposed to rather rule-abiding goals in the first component. COVID-mitigation behaviors, based on institutional prescriptions, were organized in a three-factor solution, including focused hygiene, social distancing, and risky behaviors (going out more than necessary). This structure is quite similar to other more intuitive classifications of COVID-related behaviors (Carvalho et al., 2020) and empirically driven measures of the same kind (Blagov, 2020).

Goals toward safety were positively connected to hygiene and social distancing practices and to a lower tendency to go out. On the contrary, goals to be free predicted an opposite pattern of behaviors. Multiple regression analyses showed that goals represented important predictors of behaviors, with significant unique effects above and beyond other demographic and psychological variables. This finding corroborates the idea that psychological aspects in general, and motivations in particular, are crucial elements in predicting behaviors related to COVID-19. Goals shape behaviors even more than other variables such as concern for and knowledge of the disease.

Network analysis further allowed to deepen connections between specific types of goals and behaviors. Our results showed that two goals had more direct connections with behaviors. Interestingly, these goals were somehow infused with social content. If goal #12 (breaking rules) may be seen as a reaction to the stringent and deeply unusual rules of the lockdown period, goal #24 (setting a good example) may be ascribed to a sense of social responsibility and collective interest. Behaviors directly related to goal #12 were openly rule-breaking. In contrast, all behaviors directly related to goal #24 described personal hygiene practices, with some particularly visible to others (e.g., wearing masks; goal #24 was not connected to COVID-mitigation behaviors of self-isolation and distancing, unlikely to be observable). In this sense, results suggest that social rewards might be important in shaping COVID-related behaviors, as much as other behaviors (Tamir and Hughes, 2018). This might be even more accentuated by the constant availability of reinforces in social networking websites (Rosenthal-von der Pütten et al., 2019). Crucially, our data suggest that individuals might actively seek social reinforcements by respecting the rules and thus being a good example to others or by intentionally violating them.

This study had some limitations that should be acknowledged. Albeit large, our sample was not fully representative of the Italian population. For instance, the sample included more females than males, and since the study was administered online, students and young adults were overrepresented (probably because they could access connected devices more easily). This might have introduced biases in the parameter estimates (Bethlehem, 2010). Another limitation is that behaviors were assessed through self-reports, which can be influenced by participants’ self-concept and social desirability. It could be interesting to investigate whether the emerged relationships generalize to observed behavior, as well as to critical outcomes connected to such behavior (e.g., being infected by COVID-19).

## General Discussion

Throughout two studies, we identified a set of basic motivations that individuals ascribe to respecting and violating COVID-19 mitigation norms. Moreover, we established connections between specific types of goals and behaviors relevant to COVID-19 mitigation. Our results showed that the identified goals predicted relevant behaviors above and beyond demographic variables, knowledge, impact, and concerns related to COVID-19.

The bi-dimensional structure of goals that emerged flattened more specific differences in goals (e.g., protecting self versus others), emphasizing more macroscopic aspects of safety versus freedom, which emerge as the main organizers of motivational aspects related to COVID mitigation. This probably mirrors the salience of COVID-19 pandemic and the lockdown for most laypersons. The specific unique situation probably made certain contrasts between goals particularly salient, confronting people with the issue of attributing priority either to safety or to other instances. In a way, the dimensional goal structure that emerged in the present study also recalls previous theoretical accounts on motivations in health behaviors. It has been suggested that goals “compete” for relevance (Gebhardt, 2006), behaviors being the result of the relative salience and importance of individual goals (both related and unrelated to health). The importance of self-protection goals ultimately impacts people’s health choices (Floyd et al., 2000; Milne et al., 2000). We may consider the bi-dimensional structure of COVID-related goals as a reflection of this conflict, where goals to be safe represent the primary motivation toward protection. In this sense, our network analysis approach is informative and offers fine-grained insights into the role of these competing motivations and their relative importance for health behaviors. In fact, despite bi-dimensionally organized at the higher-order level, results reveal that individual goals with a more pronounced social content may have more direct connections with behaviors, promoting adhesion to norms (setting good examples) or facilitating transgressions (breaking the rules).

Researchers agree that for encouraging appropriate behaviors related to COVID-19, as for many other health-related behaviors, informing individuals regarding what they should and should not do is not enough (Bavel et al., 2020; National Academies of Sciences, Engineering, and Medicine [NASEM], 2020). This seems to be particularly true if the recommended behaviors are in conflict with one’s ideology (Calvillo et al., 2020) or the fulfillment of one’s needs (Cantarero et al., 2020). Furthermore, as acknowledged by the WHO (World Health Organization [WHO]., 2020b), the more the pandemic becomes part of a “new normality,” the more mitigation measures become a matter of personal responsibility, rather than just something that can be simply enforced by law. We argue that individual motives will gain even greater relevance in shaping behaviors during the post-lockdown phase when institutional curfew measures are not implemented.

Knowledge of individuals’ motivations is now more crucial than ever for designing effective interventions (Wieber and Gollwitzer, 2017; Oettingen and Gollwitzer, 2018), media campaigns (National Academies of Sciences, Engineering, and Medicine [NASEM], 2020), and for the prediction of people’s behaviors related to COVID-19. Our findings suggest that motivations toward safety and freedom are potential targets for health-promotion interventions related to COVID-19. More generally, interventions promoting adherence to norms in situations of collective danger may find allies in socially oriented motivations. To encourage compliance, interventions should avoid exceedingly authoritarian messages (possibly boosting counter-reactive goals) and instead focus on providing opportunities for setting good examples in the social arena. Indeed, previous contributions suggest that rather than (or beside) enforcing, presenting mitigation behaviors as socially accepted and widely enacted behaviors is an effective way to increase adherence to rules (Webster et al., 2020; Young and Goldstein, 2021). In particular, social norms are less likely to undermine individuals’ sense of freedom (Young and Goldstein, 2021), thus reducing the risk of backfiring effects.

We believe our results may be interpreted in this light: communities could promote safety goals by showing that positive behaviors exist and are normative (which appears to be the case). Also, public messages may present mitigation behaviors as wise ways to exert one’s freedom, rather than only as obligations. These may help to build alternative ways to pursue one’s goals, tempering both safety and freedom motives. On the contrary, a sharp contrast between motivations to be free and to be safe – in this pandemic and other health-related situations – may result in polarized, potentially dangerous, behavioral responses.

## Data Availability Statement

The datasets presented in this study can be found in online repositories. The names of the repository/repositories and accession number(s) can be found below: https://osf.io/hjmyx/?view_only=75d4c3b152a7429fb22fd35fcf9119db.

## Ethics Statement

The studies involving human participants were reviewed and approved by Commissione per la Valutazione della Ricerca (CRIP), University of Milan-Bicocca. The patients/participants provided their written informed consent to participate in this study.

## Author Contributions

All authors contributed to the conception and design of the study. GC and MDS performed statistical analyses. MDS wrote the first draft of the manuscript. GC wrote sections of the manuscript. MP supervised the project. All authors contributed to manuscript revision and approved the submitted version.

## Conflict of Interest

The authors declare that the research was conducted in the absence of any commercial or financial relationships that could be construed as a potential conflict of interest.

## Footnotes

1 For details, see http://www.governo.it/it/iorestoacasa-misure-governo

2 https://www.qualtrics.com/it/

3 Preregistrations are available at https://aspredicted.org/43rr6.pdf and https://aspredicted.org/j2q8i.pdf

4 We compared demographic data of 635 excluded participants who completed at least the demographic questionnaire and of the 955 who were included in the analyses. Despite the large samples involved, the excluded participants did not present significant differences in terms of age, *t*(1628) = 1.62, *p* = 0.105, gender χ^2^(1) = 3.02, *p* = 0.092, location χ^2^(100) = 107.77, *p* = 0.281, or occupation. Participants who were excluded from the study were slightly less educated (*M* = 3.34, *SD* = 0.63) than those who completed it (*M* = 3.46, *SD* = 0.70), *t*(1628) = 3.70, *p* < 0.001. This might reflect a lower willingness to participate in an online questionnaire or a greater difficultly to complete it for less educated people.
