# Supplementary material for: Would You Rather Be Safe or Free? Motivational and Behavioral Aspects in COVID-19 Mitigation
Source: Front Psychol. 2021 May 28;12:635406. doi: 10.3389/fpsyg.2021.635406 (PMC8195459; doi:10.3389/fpsyg.2021.635406)
Supplement: Supplementary file 1 [file Data_Sheet_1.PDF]

Supplementary Material

1 Study 1: detailed software-based procedure

To perform an initial automatic classification of goals, all responses were cleared from stop-words, punctuation, and numbers. We removed a set of words that were considered as less relevant in defining the meaning of the response, such as conjunctions, determiners, pronouns, abbreviations, Italian auxiliary verbs, generic adverbs (e.g., “honestly”, “really”, “enough”), and words that did not characterize the content of the response (e.g., “goal”, “aim”, in responses such as “I am a person that…”, “my goal/aim is…”). Using the package KoRpus (Michalke, 2018) in R (R Core Team, 2020), as well as the software “TreeTagger” (Schmid, 1994, 1995), the remaining words were lemmatized. The lemmas in the corpus were inspected: Lemmas that constituted synonyms (e.g., “cause” and “provoke”), words that were close to each other in terms of meaning (e.g., “illness” and “getting sick”), as well as clear antonyms (e.g., “good” and “bad”) were considered as single units. Merging of antonyms was performed because the automatic processing did not take syntax into account. Thus, it was not able to identify expressions such as “X is not bad” and “X is good” as similar. This led to identifying 386 unique lemmas. We then generated a goal by lemma matrix, with each entry being 1 if the goal included the lemma and 0 if the goal did not. Cosine dissimilarity between each pair of goals was computed, using information about the lemmas it included. We then performed complete linkage hierarchical cluster analysis (Everitt et al., 2011) to identify groups of similar goals and employed the Dynamic Tree Cut algorithm (Langfelder et al., 2008) to automatically cut the dendrogram and identify a series of clusters of sufficiently similar goals. The initial set of 80 clusters was identified and then subject to qualitative scrutiny (see main manuscript).

Everitt, B. S., Landau, S., Leese, M., & Stahl, D. (2011). *Cluster Analysis*. John Wiley & Sons, Ltd. https://doi.org/10.1002/9780470977811

Langfelder, P., Zhang, B., & Horvath, S. (2008). Defining clusters from a hierarchical cluster tree: the Dynamic Tree Cut package for R. *Bioinformatics*, *24*(5), 719–720. https://doi.org/10.1093/bioinformatics/btm563

Michalke, M. (2018). *koRpus: An R Package for Text Analysis* (0.11-5). https://reaktanz.de/?c=hacking&s=koRpus

R Core Team. (2020). *R: A language and environment for statistical computing*. R Foundation for Statistical Computing.

Schmid, H. (1994). Probabilistic part-of-speech tagging using decision trees. *International Conference on New Methods in Language Processing*.

Schmid, H. (1995). Improvements in part-of-speech tagging with an application to German. *ACL SIGDAT-Workshop*.

2 Supplementary Tables

Table S1. Study 2: Sample distribution by location.

Area	Region	*N*	%
**North**		**753**	**75.7%**
	Lombardia[a]	483	48.5%
	Emilia Romagna[b]	143	14.4%
	Piemonte[b]	70	7.0%
	Veneto[b]	31	3.1%
	Friuli Venezia-Giulia	11	1.1%
	Trentino Alto-Adige	6	0.6%
	Liguria	6	0.6%
	Valle D’Aosta	3	0.3%
**South**		**180**	**18.1%**
	Sicilia	91	9.1%
	Calabria	47	4.7%
	Campania	13	1.3%
	Puglia	13	1.3%
	Sardegna	12	1.2%
	Abruzzo	3	0.3%
	Molise	1	0.1%
	Basilicata	0	0%
**Centre**		**62**	**6.2%**
	Lazio	29	2.9%
	Toscana	24	2.4%
	Marche	7	0.7%
	Umbria	2	0.2%

[a] High-risk region; [b] Medium/high-risk regions.

Table S2. Study 2: Loadings on the first Principal Component of the COVID-19 Goal Questionnaire.

Item		Loading
**Italian version**	**English translation**	
1. Contenere il contagio, anche se ciò può significare delle rinunce importanti	*1. Contain the contagion, even if this may imply important sacrifices*	-.67
2. Incontrare subito i miei cari (es. genitori, figli, parenti, fidanzati, ecc.), anche se ciò potrebbe comportare dei rischi	*2. Meet my loved ones immediately (e.g. parents, children, relatives, partners, etc.), even if this may involve risks*	.54
3. Cercare di non modificare la mia vita a causa dell'epidemia	*3. Try not to change my life because of the epidemic*	.41
4. Incontrare subito i miei amici, anche se ciò potrebbe comportare dei rischi	*4. Meet my friends immediately, even if this may involve risks*	.59
5. Socializzare ora, anche correndo qualche rischio (es. incontrare persone, conoscere persone nuove, avere contatto fisico)	*5. Socialize now, even taking some risks (e.g. meet people, get to know new people, have physical contact)*	.58
6. Sentirmi libero/a di fare ciò che voglio (fare ciò che mi va, senza dovermi giustificare con nessuno)	*6. Feel free to do what I want (do what I want, without having to justify myself to anyone)*	.63
7. Salvaguardare il sistema sanitario (es. prevenire o ridurre la pressione sulle strutture sanitarie, evitarne il collasso), anche se ciò significa qualche danno economico in più	*7. Safeguard the health-care system (e.g., prevent or reduce pressure on health care facilities, avoid their collapse), even if this means extra economic damage*	-.44
8. Ridurre ora i disagi dovuti alle restrizioni, anche se significa correre qualche rischio in più	*8. Reduce the inconvenience of restrictions now, even if it means taking a few more risks*	.55
9. Proteggere la mia situazione economica, anche a costo di qualche rischio per la salute	*9. Protect my economic situation, even at the cost of some risks for my health*	.54
10. Stare al sicuro (es. non correre rischi, non mettersi in pericolo, stare in salute, salvaguardarsi), anche se ciò significa annoiarsi	*10. Stay safe (e.g., don't take risks, don't put yourself in danger, stay healthy, protect yourself), even if this means getting bored*	-.67
11. Tornare a condurre una vita normale solo quando si sarà più sicuri	*11. Go back to leading a normal life only once it will be safer*	-.58
12. Fare qualcosa per il gusto di trasgredire le regole	*12. Do something for the sake of breaking the rules*	.34
13. Proteggere la mia salute, anche se questo significa per me una perdita economica	*13. Protect my health, even if this means financial losses for me*	-.58
14. Tornare a poter vedere i miei cari (es. genitori, figli, parenti, fidanzati, ecc.) solo una volta che si è più sicuri	*14. See my loved ones again (e.g. parents, children, relatives, partners, etc.) only once it is safer*	-.54
15. Divertirmi, anche se ciò significa correre qualche rischio in più	*15. Have fun, even if it means taking a few more risks*	.64
16. Fare le cose che sono importanti per me, anche se potrebbero comportare dei rischi per gli altri	*16. Do the things that are important to me, even though they may involve risks for others*	.67
17. Stare al sicuro anche rinunciando a procurarmi ciò che vorrei	*17. Stay safe, even if giving up getting what I want*	-.68
18. Stare all'aria aperta (es. fare un giro in città o al parco, passeggiare, fare sport, etc.), anche se potrebbe comportare qualche rischio di più	*18. Be outdoor (e.g. take a walk in the city or park, walk, do sports, etc.), although it may involve some extra risk*	.69
19. Salvaguardare il sistema economico (es. evitare la recessione, minimizzare i danni economici), anche se ciò significa correre qualche rischio sanitario di più	*19. Safeguard the economic system (e.g. avoid recession, minimize economic damage), even if this means taking some more risks for health*	.50
20. Proteggere la collettività, gli altri in generale, anche a scapito del mio benessere immediato (Far sì che gli altri stiano al sicuro, evitare che si ammalino)	*20. Protect the community, others in general, even at the expense of my immediate well-being (ensure others are safe, prevent them from getting sick)*	-.58
21. Rispettare norme e regole (es. rispettare la legge, le indicazioni del ministero della salute etc.)	*21. Respect norms and rules (e.g. respect the law, the directions from the Ministry of Health, etc.)*	-.64
22. Proteggere i miei cari (genitori, figli, parenti, fidanzati, ecc.), anche a scapito del mio benessere immediato (Far sì che i miei cari stiano al sicuro, evitare che si ammalino)	*22. Protect my loved ones (parents, children, relatives, partners, etc.), even at the expense of my immediate well-being (ensure my loved ones are safe, prevent them from getting sick)*	-.53
23. Dare più importanza al mio benessere che a quello generale	*23. Give more importance to my well-being than to the general well-being*	.40
24. Dare il buon esempio agli altri	*24. Set a good example to others*	-.52
25. Tornare a poter vedere i miei amici solo una volta che si è più sicuri.	*25. See my friends again only once it is safer*	-.62

Table S3. Study 2: Loadings on the first Principal Component of the COVID-19 Behaviors Questionnaire.

Item		**Loading**
**Italian version**	**English translation**	
1. Restare a casa, uscire di casa solo per esigenze lavorative, motivi di salute e necessità	*1. Stay at home, leave home only for work, health and necessity reasons*	.18
2. Uscire di casa (ad es., per fare la spesa o andare in farmacia), anche se non strettamente necessario	*2. Leave the house (e.g., to go shopping or to the pharmacy), even if not strictly necessary*	-.34
3. Uscire di casa per motivi consentiti dalla normativa e approfittarne per allontanarsi e fare due passi	*3. Leave home for reasons permitted by law and take advantage of it to get away and go for a walk*	-.34
4. Uscire molto spesso di casa per fare sport all'aperto	*4. Go out of the house very often for outdoor sports*	-.27
5. Cercare delle ragioni qualsiasi per uscire di casa, anche se non veramente necessario	*5. Look for any reason to leave the house, even if it's not really necessary*	-.36
6. Incontrare persone (escluse le persone con cui si convive)	*6. Meet people (excluding people you live with)*	-.34
7. Lavare frequentemente le mani	*7. Wash my hands frequently*	.47
8. Pulire le mani con gel o salviette igienizzanti, quando non si dispone di acqua e sapone	*8. Clean my hands with gel or sanitizing wipes when I do not have soap and water*	.48
9. Evitare assembramenti di persone	*9. Avoid people gatherings*	.54
10. Evitare di creare occasioni di incontro in casa o fuori, con amici, parenti e/o vicini	*10. Avoid creating opportunities to meet at home or outside, with friends, relatives and/or neighbors*	.50
11. Mantenere, nei contatti sociali, una distanza interpersonale di almeno un metro (escluse le persone con cui si convive)	*11. Maintain, in social contacts, an interpersonal distance of at least one meter (excluding people with whom you live together).*	.53
12. Evitare abbracci e/o strette di mano (tranne che con le persone con cui si convive)	*12. Avoid hugs and/or handshakes (except with people you live with)*	.59
13. Igiene respiratoria (starnutire e/o tossire in un fazzoletto evitando il contatto delle mani con le secrezioni respiratorie)	*13. Respiratory hygiene (sneeze and/or cough in a handkerchief avoiding hand contact with respiratory secretions)*	.53
14. Non toccarsi occhi, naso e bocca con le mani, senza averle lavate prima	*14. Do not touch my eyes, nose, and mouth with hands without washing them in advance*	.53
15. Evitare il contatto ravvicinato con persone che soffrono di infezioni respiratorie acute	*15. Avoid close contact with people suffering from acute respiratory infections*	.44
16. Evitare l'uso promiscuo di bottiglie e bicchieri	*16. Avoid sharing bottles or glasses*	.52
17. Pulire le superfici con disinfettanti a base di cloro o alcol	*17. Clean surfaces with chlorine or alcohol-based disinfectants*	.49
18. Usare i guanti e/o la mascherina fuori casa (incluse protezioni alternative come sciarpe o bandane)	*18. Use gloves and/or mask when outside (including alternative protections such as scarves or bandanas)*	.49
